# From global to national: The role of urban agglomerations in China’s new development paradigm

**DOI:** 10.1371/journal.pone.0305594

**Published:** 2024-06-17

**Authors:** Chang-chun Gao, Si-qi Chang, Ying-su Wang

**Affiliations:** Glorious Sun School of Business and Management, Donghua University, Shanghai, 200051, China; East China Normal University, CHINA

## Abstract

Urban agglomerations (UAs), which serve as pivotal hubs for economic and innovative convergence, play a crucial role in enhancing internal circulation and strengthening external linkages. This study utilizes the China city-level multi-regional input-output tables, incorporating the Dagum Gini coefficient and kernel density estimation methods, to perform a thorough quantitative analysis. Disparities within the national and global value chains ("dual value chains") of Chinese UAs from 2012 to 2017 were assessed. Additionally, the logarithmic mean Divisia index (LMDI) method was applied to disaggregate the drivers of both national and global intermediate inputs (NII and GII). The study’s key findings include the following: (1) The national value chain (NVC) within UAs exhibits robust growth, contrasting with the decline in the global value chain (GVC). (2) The inter-UA disparity contribution rate significantly surpasses the combined rates of intra-UA contribution and super-variation density. (3) Distinct evolutionary peak trends are discerned among various UAs within the "dual value chains", highlighting diverse spatial polarization characteristics and expansiveness. (4) The growth of the NVC has transitioned from a negative to a positive impact on NII, while the decline in GVC has substantially counteracted GII growth. Economic and demographic factors notably drive positive improvements in both NII and GII, whereas the efficiency of outflows presents a negative driving effect. Based on these findings, this study offers strategic recommendations to facilitate the effective integration of UAs into the new development paradigm, thereby providing a scientific basis for related decision-making processes.

## Introduction

As globalization accelerates, urban agglomerations (UAs) increasingly become pivotal nodes for economic growth and innovation, playing an essential role in the evolution of economic development patterns [1–3]. This global trend is keenly observed in China, where the economy has transitioned to a "new normal", shifting from rapid growth to prioritizing high-quality development [4–6]. In this evolved economic landscape, UAs are not merely engines of economic development and urbanization [7–9]; they also serve as crucial connectors between domestic and international economies [10–12], fostering international cooperation and competition [13–15].

Building on this foundational understanding, a new development paradigm emerges where the domestic and international markets can boost each other, with the domestic market as the mainstay [16]. Within this framework, UAs have gained a strategic prominence [17], becoming instrumental in driving regional economic development [18] and social progress. Reflecting their strategic importance, China’s 11th through 14th Five-Year Plans have consistently underscored UAs as central to advancing the new urbanization agenda. Similarly, reports from the 17th through 20th National Congresses of the Communist Party of China have recognized UAs as burgeoning epicenters of economic growth. Therefore, an in-depth exploration of the role of UAs within national and global value chains ("dual value chains") and their contribution to China’s new development paradigm is critically significant [19]. This exploration is not just relevant for understanding the current economic transformation [20,21] in China but also for gauging its impact on the global economic landscape [22], making the study of UAs a key area of interest in the field of economic development.

The research presented in this paper intersects with three strands of literature, the first of which concerns economic transformation under the new development paradigm. Scholars have primarily discussed this paradigm from various perspectives, including its developmental framework, key issues, and policy implications. Lin & Wang (2022) [23] provided a detailed explanation of China’s dual circulation development paradigm from the perspective of new structural economics and examined its theoretical underpinnings. Javed et al. (2023) [24] analyzed critical issues that could lead China’s economy towards dual circulation or a new round of strategic reforms, discussing the significance of these issues. Lin (2021) [25] interpreted the new development strategy through the interplay of domestic circulation, international circulation, and the mutual reinforcement between them, indicating potential pathways for realization.

The second strand focuses on the role of UAs in promoting high-quality development. This segment of literature examines UAs as economic and innovation hubs, delving into the specific mechanisms and impacts they have on high-quality development, including industrial upgrading, technological innovation, and regional integration. Song et al. (2021) [26] investigated the reasons behind the slowdown in industrial carbon dioxide emissions growth since China’s economy entered the "new normal" phase in 2012. Zhang et al. (2020) [27] discussed the spatial structure of UAs under the influence of high-speed rail construction using social network analysis. Xie et al. (2021) [28] highlighted the role of financial agglomeration in enhancing the green total factor productivity of Chinese cities. Han et al. (2021) [29] conducted a spatiotemporal analysis of the coordination between economic development, resource utilization, and environmental quality in the Beijing-Tianjin-Hebei UA. Sun et al. (2023) [30] discussed the effects of digital finance on carbon productivity within UAs, underscoring the notable spatial spillover effects.

The third strand pertains to the measurement of "dual value chains," primarily focusing on the provincial level. Meng et al. (2013) [31] developed a framework that endogenously embeds a country’s domestic interregional input-output tables into the international input-output model to measure domestic linkages. Several scholars have applied global value chain (GVC) measurements to national value chain (NVC) from various perspectives. Su (2016) [32] extended the framework of Koopman et al. (2014) [33] for decomposing the sources of export value at the national level to the regional level within a country. Li & Pan [34] integrated "dual value chains" under one framework by building upon the models of Koopman et al. (2014) [33] and Wang et al. (2013) [35].

Overall, research on the theoretical essence and implementation pathways of the new development paradigm has matured, with studies on the quantification of value chain integration levels becoming increasingly rich. However, these analyses, often grounded in provincial-level data, overlook the substantial differences in factor endowments and industrial structures between regions. The emergence of UAs has transcended provincial boundaries, reshaping the spatial units of economic activity [36,37]. This indicates that there is still room for improvement in research on spatial disparities and structural decomposition. To some extent, this may lead to inaccuracies in the positioning of UAs, thereby hindering the formulation of targeted measures for better integration into the new development paradigm. Moreover, there is a scarcity of literature that quantitatively assesses "dual value chains" as a driving factor, which is a critical area [38] for providing pathway references to effectively advance the new development paradigm.

Therefore, analyzing the status of different UAs within "dual value chains" and unveiling the intra-UA differences is urgent. It necessitates conducting research on the distinctive characteristics and dynamic evolution patterns of "dual value chains" among various UAs. Furthermore, with the fluctuations of economic globalization and the uncertainty of the international environment, the challenges and opportunities faced by Chinese UAs are changing. This requires a more precise evaluation and understanding of the impact of UAs as driving factors within dual value chains on domestic and international intermediate inputs (NII/GII). This study aims to reveal the positioning of Chinese UAs within dual value chains under the new development paradigm, providing a theoretical reference for advancing the realization of this new paradigm.

The remainder of our study is structured as follows: Section 2 details the methodologies and data sources employed in this study. Section 3 presents the results and discussion. Finally, Section 4 outlines the conclusions.

## Methods and data

### Intercity multi-regional input-output model

The concept of regional outflow (*OF*) within a specific domestic area is defined as the aggregate of intermediate and final products flowing from that region to areas external to it, as described by Koopman et al. (2014) [33]. This encompasses both outflows to other domestic regions and exports to foreign destinations. Considering the case of a nation with *G* regions and *N* industries, the total output of the domestic region *r* (where *r* = 1,2,⋯*G*) can be expressed as follows:

$$X^{r}=A^{r1}X^{1}+A^{r2}X^{2}+\ldots +A^{rG}X^{G}+DY^{r1}+DY^{r2}+\ldots +DY^{\mathrm{rG}}+e^{r}$$ (1)

In this context, *X*^*r*^ represents the N×1 gross output vector for region *r*, *DY*^*rs*^ signifies the N×1 domestic final demand vector from region *r* to domestic region *s*, *e*^*r*^ represents the N×1 total output vector for region *r* exported to foreign destinations (comprising both intermediate and final products), and *A*^*sr*^ represents the N×N input-output coefficient matrix denoting the domestic region *r*’s demand for intermediate products from region *s*, rewritten in matrix form as:

$$\left[\begin{array}{c} X^{1}\\ X^{2}\\ \vdots \\ X^{G} \end{array}]=[\begin{array}{cccc} A^{11} & A^{12} & \cdots & A^{1G}\\ A^{21} & A^{22} & \cdots & A^{2G}\\ \vdots & \vdots & \ddots & \vdots \\ A^{G1} & A^{G2} & \cdots & A^{GG} \end{array}][\begin{array}{c} X^{1}\\ X^{2}\\ \vdots \\ X^{G} \end{array}]+[\begin{array}{c} \sum_{r=1}^{G}DY^{1r}+e^{1}\\ \sum_{r=1}^{G}DY^{2r}+e^{2}\\ \vdots \\ \sum_{r=1}^{G}DY^{Gr}+e^{G} \end{array}]=[\begin{array}{cccc} B^{11} & B^{12} & \cdots & B^{1G}\\ B^{21} & B^{22} & \cdots & B^{2G}\\ \vdots & \vdots & \ddots & \vdots \\ B^{G1} & B^{G2} & \cdots & B^{GG} \end{array}]\times [\begin{array}{c} \sum_{r=1}^{G}DY^{1r}+e^{1}\\ \sum_{r=1}^{G}DY^{2r}+e^{2}\\ \vdots \\ \sum_{r=1}^{G}DY^{Gr}+e^{G} \end{array}\right]$$ (2)

where *B*^*sr*^ represents the block Leontief inverse matrix. As derived from Eq (2):

$$X^{r}=\sum_{t=1}^{G}B^{rt}(\sum_{u=1}^{G}DY^{tu}+e^{t})$$ (3)

The outflow from region *s* to region *r* is defined as follows:

$$OF^{sr}=A^{sr}X^{r}+DY^{sr}$$ (4)

The total outflow from region *s* to both domestic regions and foreign destinations is defined as follows:

$$OF^{s*}=\sum_{r\neq s}^{G}(A^{sr}X^{r}+DY^{sr})+e^{s}$$ (5)

By combining Eqs (3) and (4), we derive:

$$\begin{array}{c} OF^{sr}=A^{sr}B^{rr}DY^{rr}+A^{sr}\sum_{t\neq s,r}^{G}B^{rt}DY^{tt}+A^{sr}B^{rr}\sum_{t\neq s,r}^{G}DY^{rt}+A^{sr}\sum_{t\neq s,r}^{G}B^{rt}\sum_{u\neq s,t}^{G}DY^{tu}+A^{sr}B^{rr}DY^{rs}\\ \;+Asr\sum_{t\neq s,r}^{G}B^{rt}DY^{ts}+A^{sr}B^{rs}DY^{ss}+A^{sr}\sum_{t\neq s}^{G}B^{rs}DY^{st}+A^{sr}B^{rr}e^{r}+A^{sr}B^{rs}e^{s}+A^{sr}\sum_{t\neq s,r}^{G}B^{rt}e^{t}+DY^{sr} \end{array}$$ (6)

Based on Eqs (1) and (5), the following relationship is derived:

$$X^{r}=A^{rr}X^{r}+DY^{rr}+OF^{r*}={\left(I-A^{rr}\right)}^{-1}DY^{rr}+{\left(I-A^{rr}\right)}^{-1}OF^{r*}$$ (7)

Taking $L^{rr}\equiv {\left(I-A^{rr}\right)}^{-1}$ *L*^*rr*^ ≡(*I*−*A*^*rr*^)^−1^ as the local Leontief inverse matrix, intermediate products can be classified into two distinct categories based on the regions in which they are utilized:

$$A^{sr}X^{r}=A^{sr}L^{rr}DY^{rr}+AS^{rr}L^{rr}OF^{r*}$$ (8)

Let *V*^*s*^ be the 1×Nvector representing the direct value-added coefficients for region *s*, and let *M*^*s*^ be the 1×N import coefficient vector for region *s*. We define:

$$VAS=[\begin{array}{cccc} V^{1}B^{11} & V^{1}B^{12} & \cdots & V^{1}B^{1G}\\ V^{2}B^{21} & V^{2}B^{22} & \cdots & V^{2}B^{2G}\\ \vdots & \vdots & \ddots & \vdots \\ V^{G}B^{G1} & V^{G}B^{G2} & \cdots & V^{G}B^{GG} \end{array}]$$ (9)

$$MS=[\begin{array}{cccc} M^{1}B^{11} & M^{1}B^{12} & \cdots & M^{1}B^{1G}\\ M^{2}B^{21} & M^{2}B^{22} & \cdots & M^{2}B^{2G}\\ \vdots & \vdots & \ddots & \vdots \\ M^{G}B^{G1} & M^{G}B^{G2} & \cdots & M^{G}B^{GG} \end{array}]$$ (10)

In Eq (9), the elements on the diagonal of *VAS* represent the regional value-added shares in the outflows, while the off-diagonal elements in each column represent the value-added shares from one domestic region to another in the outflows. In Eq (10), the elements on the diagonal of *MS* represent the marginal share of imports within each region’s outflows (foreign component share), and the off-diagonal elements in each column represent the marginal share of imports acquired by a region indirectly through domestic interregional trade (indirect foreign component share). The sum of each column in *MS* represents the marginal share of foreign components induced by a region’s trade outflows. Based on the relationships of the value-added coefficient vector, import coefficient vector, and Leontief inverse matrix, we have:

$$\sum_{r=1}^{G}(V^{r}+M^{r})B^{rs}=u$$ (11)

Within the framework of value-added accounting and taking into account the following symbols, when matrices *A* and *B* have identical dimensions, the notation *A#B* represents elementwise multiplication of *A* and *B*, such that *C* ≡ *A#B* is equivalent to *C*(*i*,*j*)≡*A*(*i*,*j*)*B(*i*,*j*). As a result, following Eqs (6), (8) and (11), the outflows from region *s* to region *r* can be decomposed as described below:

$$\begin{array}{l} OF^{sr}{=}_{1}{\left(V^{s}B^{ss}\right)}^{T}\#DY^{sr}{+}_{2}{\left(V^{s}L^{ss}\right)}^{T}\#(A^{sr}B^{rr}DY^{rr}){+}_{3}{\left(V^{s}L^{ss}\right)}^{T}\#(A^{sr}\sum_{t\neq s,r}^{G}B^{rt}DY^{tt})\\ \;{+}_{4}{\left(V^{s}L^{ss}\right)}^{T}\#(A^{sr}B^{rr}\sum_{t\neq s,r}^{G}DY^{rt}){+}_{5}{\left(V^{s}L^{ss}\right)}^{T}\#(A^{sr}\sum_{t\neq s,r}^{G}B^{rt}\sum_{u\neq s,t}^{G}DY^{tu})\\ \;{+}_{6}{\left(V^{s}L^{ss}\right)}^{T}\#(A^{sr}B^{rr}DY^{rs}){+}_{7}{\left(V^{s}L^{ss}\right)}^{T}\#(A^{sr}\sum_{t\neq s,r}^{G}B^{rt}DY^{ts}){+}_{8}{\left(V^{s}L^{ss}\right)}^{T}\#(A^{sr}B^{rs}DY^{ss})\\ \;{+}_{9}{\left(V^{s}L^{ss}\right)}^{T}\#(A^{sr}\sum_{t\neq s}^{G}B^{rs}DY^{st}){+}_{10}{\left(V^{s}B^{ss}-V^{s}L^{ss}\right)}^{T}\#(A^{sr}X^{r}){+}_{11}{\left(V^{s}L^{ss}\right)}^{T}\#(A^{sr}B^{rr}e^{r})\\ \;{+}_{12}{\left(V^{s}L^{ss}\right)}^{T}\#(A^{sr}B^{rs}e^{s}){+}_{13}{\left(V^{s}L^{ss}\right)}^{T}\#(A^{sr}\sum_{t\neq s,r}^{G}B^{rt}e^{t}){+}_{14}{\left(V^{s}B^{rs}\right)}^{T}\#DY^{sr}\\ \;{+}_{15}{\left(V^{r}B^{rs}\right)}^{T}\#(A^{sr}L^{rr}DY^{rr}){+}_{16}{\left(V^{r}B^{rs}\right)}^{T}\#(A^{sr}L^{rr}OF^{r*}){+}_{17}{\left(\sum_{t\neq s,r}^{G}V^{t}B^{ts}\right)}^{T}\#DY^{sr}\\ \;{+}_{18}{\left(\sum_{t\neq s,r}^{G}V^{t}B^{ts}\right)}^{T}\#(A^{sr}L^{rr}DY^{rr}){+}_{19}{\left(\sum_{t\neq s,r}^{G}V^{t}B^{ts}\right)}^{T}\#(A^{sr}L^{rr}OF^{r*}){+}_{20}{\left(M^{s}B^{ss}\right)}^{T}\#DY^{sr}\\ \;{+}_{21}{\left(M^{s}L^{ss}\right)}^{T}\#(A^{sr}B^{rr}DY^{rr}){+}_{22}{\left(M^{s}L^{ss}\right)}^{T}\#(A^{sr}\sum_{t\neq s,r}^{G}B^{rt}DY^{tt}){+}_{23}{\left(M^{s}L^{ss}\right)}^{T}\#(A^{sr}B^{rr}\sum_{t\neq s,r}^{G}DY^{rt})\\ \;{+}_{24}{\left(M^{s}L^{ss}\right)}^{T}\#(A^{sr}\sum_{t\neq s,r}^{G}B^{rt}\sum_{u\neq s,r}^{G}DY^{tu}){+}_{25}{\left(M^{s}L^{ss}\right)}^{T}\#(A^{sr}B^{rr}DY^{rs})\\ \;{+}_{26}{\left(M^{s}L^{ss}\right)}^{T}\#(A^{sr}\sum_{t\neq s,r}^{G}B^{rt}DY^{ts}){+}_{27}{\left(M^{s}L^{ss}\right)}^{T}\#(A^{sr}B^{rs}DY^{ss})\\ \;{+}_{28}{\left(M^{s}L^{ss}\right)}^{T}\#(A^{sr}\sum_{t\neq s}^{G}B^{rs}DY^{st}){+}_{29}{\left(M^{s}B^{ss}-M^{s}L^{ss}\right)}^{T}\#(A^{sr}X^{r})\\ \;{+}_{30}{\left(M^{s}L^{ss}\right)}^{T}\#(A^{sr}B^{rr}e^{r}){+}_{31}{\left(M^{s}L^{ss}\right)}^{T}\#(A^{sr}B^{rs}e^{s}){+}_{32}{\left(M^{s}L^{ss}\right)}^{T}\#(A^{sr}\sum_{t\neq s,r}^{G}B^{rt}e^{t})\\ \;{+}_{33}{\left(M^{r}B^{rs}\right)}^{T}\#DY^{sr}{+}_{34}{\left(M^{r}B^{rs}\right)}^{T}\#(A^{sr}L^{rr}DY^{rr}){+}_{35}{\left(M^{r}B^{rs}\right)}^{T}\#(A^{sr}L^{rr}OF^{r*})\\ \;{+}_{36}{\left(\sum_{t\neq s,r}^{G}M^{t}B^{ts}\right)}^{T}\#DY^{sr}{+}_{37}{\left(\sum_{t\neq s,r}^{G}M^{t}B^{ts}\right)}^{T}\#(A^{sr}L^{rr}DY^{rr}){+}_{38}{\left(\sum_{t\neq s,r}^{G}M^{t}B^{ts}\right)}^{T}\#(A^{sr}L^{rr}OF^{r*}) \end{array}$$ (12)

Building upon this foundation, the total outflows from region *s* can be decomposed into the following components:

$$\begin{array}{l} OF^{s*}=\sum_{r\neq s}^{G}OF^{sr}+e^{s}\\ \;{=}_{1-38}\sum_{r\neq s}^{G}OF^{sr}{+}_{39}{\left(V^{s}B^{ss}\right)}^{T}e^{s}+40{\left(\sum_{r\neq s}^{G}V^{r}B^{rs}\right)}^{T}\#e^{s}{+}_{41}{\left(M^{s}B^{ss}\right)}^{T}\#e^{s}{+}_{42}{\left(\sum_{r\neq s}^{G}M^{r}B^{rs}\right)}^{T}\#e^{s} \end{array}$$ (13)

In Eqs (12) and (13), the subscripts denoting each term on the right-hand side are indicated as sequential numbers in the lower-left corner.

Employing this framework and referring to Wang et al. (2013) as a guide [35], we conducted an investigation to calculate the positions and participation indices of different Chinese UAs within both the NVC and GVC.


$$NVC_{i}=Term(2+\ldots +5+14+15+17+18+40)/OF^{s*}=NII/OF^{s*}$$ (14)

$$GVC_{i}=Term(11+13+20+\ldots +27+30+32+33+34+36+37+39+41+42)/OF^{s*}=GII/OF^{s*}$$ (15)

In Eq (14), *NII* denotes the national intermediate inputs, referring to the value-added of a region as intermediate inputs flowing into other domestic regions and the domestic intermediate inputs used in the total outflows of that region. Similarly, in Eq (15), *GII* represents the global intermediate inputs, encompassing the value-added of a region as intermediate inputs flowing into foreign countries and the foreign intermediate inputs used in the total outflows of that region.

### Dagum Gini coefficient

Dagum’s decomposition approach for the Gini coefficient is adept at dissecting overall disparities into intra-regional disparities, inter-regional disparities, and super-variation density, thereby offering a more transparent elucidation of spatial disparity sources [39]. This method effectively overcomes the limitations of traditional regional disparity measurement techniques, especially those not accounting for overlapping data issues, thus enabling a more refined identification of regional disparity sources [40]. In this study, Dagum’s decomposition was applied to scrutinize disparities and their origins within the "dual value chains" of 18 urban agglomerations in China. The methodology for computing the Dagum Gini coefficient is delineated in Eq (16):

$$G=\sum_{j=1}^{k}\sum_{h=1}^{k}\sum_{i=1}^{n_{j}}\sum_{r=1}^{n_{k}}\left|y_{ji}-y_{hr}\right|/2n^{2}\bar{y}$$ (16)

In Eq (14), *G* represents the overall disparity; *k* is the number of UAs; *n* is the number of administrative units within each UA; *j*(*h*) denotes the UA; and *i*(*r*) represents the administrative unit, where *y*_*ji*_ and *y*_*hr*_ denote the NVC/GVC of administrative unit *i*(*r*) in the *j*(*h*)^*th*^ UA, respectively; and $\bar{y}$ is the average value of NVC/GVC for all administrative units. *G* can be decomposed into the intra-UA disparity *G*_*w*_, inter-UA disparity *G*_*nb*_, and super-variation density *G*_*t*_. The calculation methods for these three components are shown in Eqs (17), (18) and (19), respectively.


$$Gw=\sum_{j=1}^{k}G_{jj}P_{j}S_{j}$$ (17)

$$G_{nb}=\sum_{j=2}^{k}\sum_{h=1}^{j-1}G_{jh}(P_{j}S_{h}+P_{h}S_{j})D_{jh}$$ (18)

$$G_{t}=\sum_{j=2}^{k}\sum_{h=1}^{j-1}G_{jh}(P_{j}S_{h}+P_{h}S_{j})(1-D_{jh})$$ (19)

Among them, *P*_*j*_ = *n*_*j*_/*n*; $S_{j}=n_{j}{\bar{y}}_{j}/n\bar{y}$. *D*_*jh*_ represents the interactive impact of NVC (GVC) between UAs *j* and *h*; *d*_*jh*_ refers to the difference in NVC (GVC) between administrative units, indicating the mathematical expectation of the sum of all samples in UAs *j* and *h* where *y*_*hr*_−*y*_*ji*_>0; and *F*_*j*_(*F*_*h*_) represents the cumulative density distribution function of UAs *j*(*h*).


$$D_{jh}=\left(d_{jh}-P_{jh}\right)/\left(d_{jh}+P_{jh}\right)$$ (20)

$$d_{jh}=\int_{0}^{\infty }dFj(y)\int_{0}^{y}\left(y-x\right)dF_{h}(x)$$ (21)

$$P_{jh}=\int_{0}^{\infty }dF_{h}(y)\int_{0}^{y}\left(y-x\right)dF_{j}(x)$$ (22)

### Kernel density estimation

Kernel density estimation utilizes a continuous density curve to characterize the distribution shape of a random variable, reflecting the distribution’s location, form, and spread; this method is widely used in spatial nonequilibrium analysis [41]. Assuming that the density function of the random variable *X* is:

$$f(x)=\frac{1}{mb}\sum_{i=1}^{m}K(\frac{X_{i}-x}{b})$$ (23)

*m* denotes the number of observations, *b* represents the bandwidth, *K*(*) is the kernel density function, *X*_*i*_ are the independently distributed sample observations, and *x* is the mean. This study employs the Gaussian kernel density for estimation, expressed as follows:

$$K(X)=\frac{1}{\sqrt{2\pi }}\exp(-\frac{x^{2}}{2})$$ (24)

### LMDI

The LMDI method is renowned for its absence of residuals, its analytical straightforwardness, and its additive properties [42–44]. This approach is particularly suited for the meticulous decomposition of specific factors [45,46].

In our study, the LMDI method was employed to disaggregate the drivers of both national and global intermediate inputs (NII and GII). Acknowledging the diverse characteristics of UAs, the decomposition of the NII and GII for each UA is structured as follows:

$$NII_{i}=\frac{NII_{i}}{OF_{i}}\times \frac{OF_{i}}{GDP_{i}}\times \frac{GDP_{i}}{POP_{i}}\times POP_{i}=NVC_{i}\times OE_{i}\times PGDP_{i}\times POP_{i}$$ (25)

$$GII_{i}=\frac{GII_{i}}{OF_{i}}\times \frac{OF_{i}}{GDP_{i}}\times \frac{GDP_{i}}{POP_{i}}\times POP_{i}=GVC_{i}\times OE_{i}\times PGDP_{i}\times POP_{i}$$ (26)

The meanings and units of the variables in Eqs (25) and (26) are shown in Table 1.

**Table 1:** Variables used in the LMDI analysis.

Variable	Symbol	Meaning	Unit
Global value chain	*GVC*_*i*_	Global value chain embeddedness in i-th UA	-
National value chain	*NVC*_*i*_	National value chain embeddedness in i-th UA	-
Outflow efficiency	*OE*_*i*_	Regional outflows per unit of GDP in i-th UA	-
Per capita GDP	*PGDP*_*i*_	Per capita GDP of i-th UA	10^3^ CNY/Person
Population size	*PIP*_*i*_	Population size of i-th UA	10^6^ Person

From time *t* to *T*, the variation in *GII*_*i*_ and *NII*_*i*_ can be expressed as the result of the combined effect of four driver factors:

$$\Delta NII_{i}=NII_{i,T}-NII_{i,t}=\Delta NVC_{i}+\Delta OE_{i}^{NII}+\Delta PGDP_{i}^{NII}+\Delta POP_{i}^{NII}$$ (27)

$$\Delta GII_{i}=GII_{i,T}-GII_{i,t}=\Delta GVC_{i}+\Delta OE_{i}^{GII}+\Delta PGDP_{i}^{GII}+\Delta POP_{i}^{GII}$$ (28)

The abovementioned terms can be calculated as follows:

$$\Delta NVC_{i}=W_{i}^{NII}.\ln(\frac{NVC_{i,T}}{NVC_{i,t}})$$ (29)

$$\Delta OE_{i}^{NII}=W_{i}^{NII}.\ln(\frac{OE_{i,T}^{NII}}{OE_{i,t}^{NII}})$$ (30)

$$\Delta PGDP_{i}^{NII}=W_{i}^{NII}.\ln(\frac{PGDP_{i,T}^{NII}}{PGDP_{i,t}^{NII}})$$ (31)

$$\Delta POP_{i}^{NII}=W_{i}^{NII}.\ln(\frac{POP_{i,T}^{NII}}{POP_{i,t}^{NII}})$$ (32)

$$\Delta GVC_{i}=W_{i}^{GII}.\ln(\frac{GVC_{i,T}}{GVC_{i,t}})$$ (33)

$$\Delta OE_{i}^{GII}=W_{i}^{GII}.\ln(\frac{OE_{i,T}^{GII}}{OE_{i,t}^{GII}})$$ (34)

$$\Delta PGDP_{i}^{GII}=W_{i}^{GII}.\ln(\frac{PGDP_{i,T}^{GII}}{PGDP_{i,t}^{GII}})$$ (35)

$$\Delta POP_{i}^{GII}=W_{i}^{GII}.\ln(\frac{POP_{i,T}^{GII}}{POP_{i,t}^{GII}})$$ (36)

Where $W_{i}^{NII}=\frac{NII_{i,T}-NII_{i,t}}{\ln NII_{i,T}-\ln NII_{i,t}}$ and $W_{i}^{GII}=\frac{GII_{i,T}-GII_{i,t}}{\ln GII_{i,T}-\ln GII_{i,t}}$, $W_{i}^{NII}$ and $W_{i}^{GII}$ are called logarithmic mean weight.

### Data and sources

The dataset for this research is derived from the Carbon Emission Accounts and Datasets for emerging economies (CEADs) [47], which provides city-level multi-regional input-output tables for the years 2012, 2015, and 2017, encompassing 313 administrative units in mainland China. Additionally, data pertinent to economic growth, population dynamics, and other key drivers were sourced from the EPSDATA database and the statistical yearbooks of each administrative unit. To align with the geographical scope of China’s Urban Agglomeration Development Plans and the coverage of CEADs, this study focuses on 210 administrative units across 18 UAs. These administrative units collectively represent more than 91% of the areas designated in the UA plans, thereby bolstering the representativeness and precision of the study’s findings. The 18 UAs analyzed included the following: Central and Southern Liaoning (CSL), Harbin-Changchun (HC), Beijing-Tianjin-Hebei (BTH), Shandong Peninsula (SP), Yangtze River Delta (YRD), Guangdong-Fujian-Zhejiang coastal region (GFZ), Pearl River Delta (PRD), Central Shanxi (CS), Central Plains (CP), the middle reaches of the Yangtze River (MYR), Hohhot-Baotou-Ordos-Yulin (HBOY), Chengdu-Chongqing (CC), Central Guizhou (CG), Guanzhong Plain (GP), Beibu Gulf (BB), the Yellow River in Ningxia (NYR), Lanzhou-Xining (LX), and the northern slope of the Tianshan Mountains (NSTM). Central Yunnan was excluded from the analysis due to substantial data limitations.

## Results and discussion

### UAs disparities and decomposition in "dual value chains"

#### Overall and intra-UA disparity analysis

**Fig 1:**
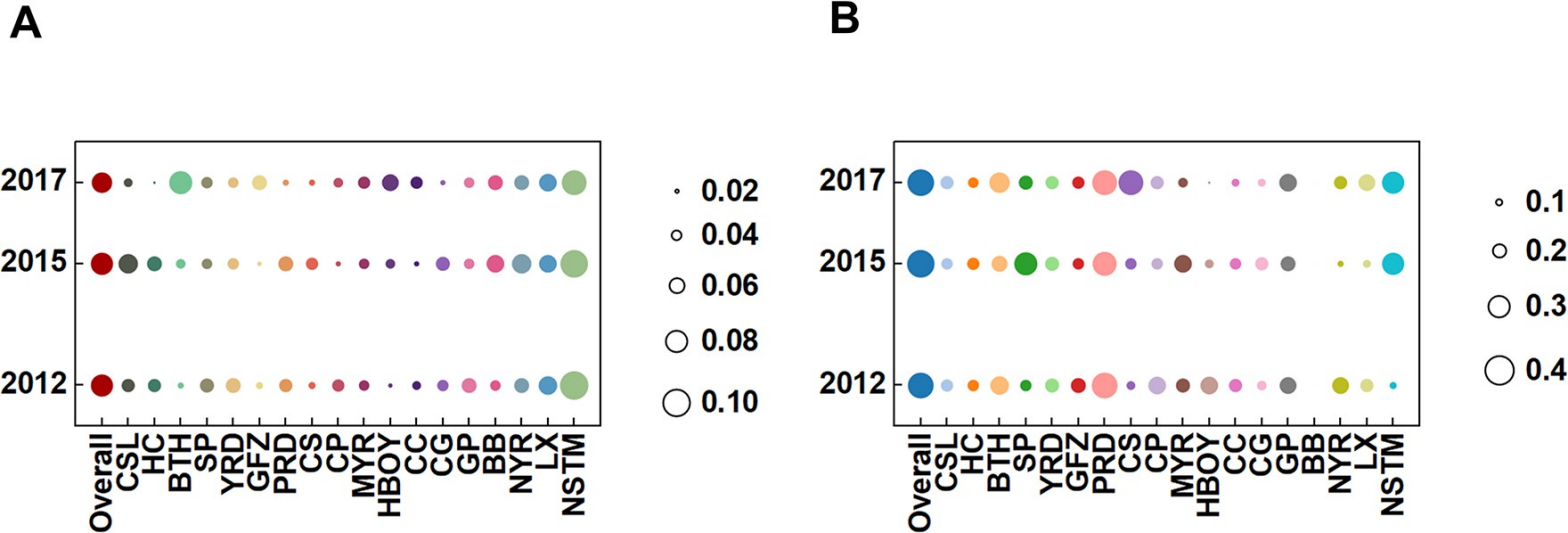
Analysis of overall and intra-UA disparities in Chinese UAs. (A) NVC analysis. (B) GVC analysis.

Fig 1A demonstrates that the overall disparity in the NVC in China was 0.07826 in 2012, which marginally decreased to 0.07811 in 2015 and further declined to 0.07276 by 2017. This consistent reduction indicates a trend toward more equitable development of NVC across the nation. Examining intra-UA disparities, with BTH and PRD as exemplars, reveals contrasting patterns. In BTH, the NVC disparity escalated from 0.02193 in 2012 to 0.08051 in 2017, suggesting an increase in economic concentration or rapid development in specific sectors. Conversely, in the PRD, the disparity decreased from 0.04563 in 2012 to 0.02273 in 2017, which was attributed largely to industrial structure optimization and enhanced regional economic integration. These variations highlight the multifaceted and dynamic nature of NVC disparities in Chinese urban agglomerations shaped by regional policies, industrial development levels, and regional integration processes.

As shown in Fig 1B, the overall disparity in the GVC exhibited an initial increase from 0.3363 in 2012 to 0.3671 in 2015, followed by a reduction to 0.3485 in 2017. Unlike in NVC, in GVC, the overall disparity trajectory reflects global economic shifts and China’s evolving role within the GVC. Intra-UA disparity analysis reveals diverse trends. BTH’s disparity significantly rose to 0.2713 in 2017, indicative of growing economic imbalances associated with rapid urbanization and industrial concentration. Conversely, CS experienced a marked increase in disparity between 2015 and 2017, suggesting an intensification of economic development imbalances. The PRD consistently displayed high disparity levels, underscoring its pivotal role in the GVC and ongoing internal economic imbalances. These findings elucidate the dynamic nature of GVC across different Chinese urban agglomerations and their inherent disparities, arising from unique development approaches, industrial structure imbalances, regional economic interactions, and global economic influences.

#### Inter-UA disparity analysis

**Fig 2:**
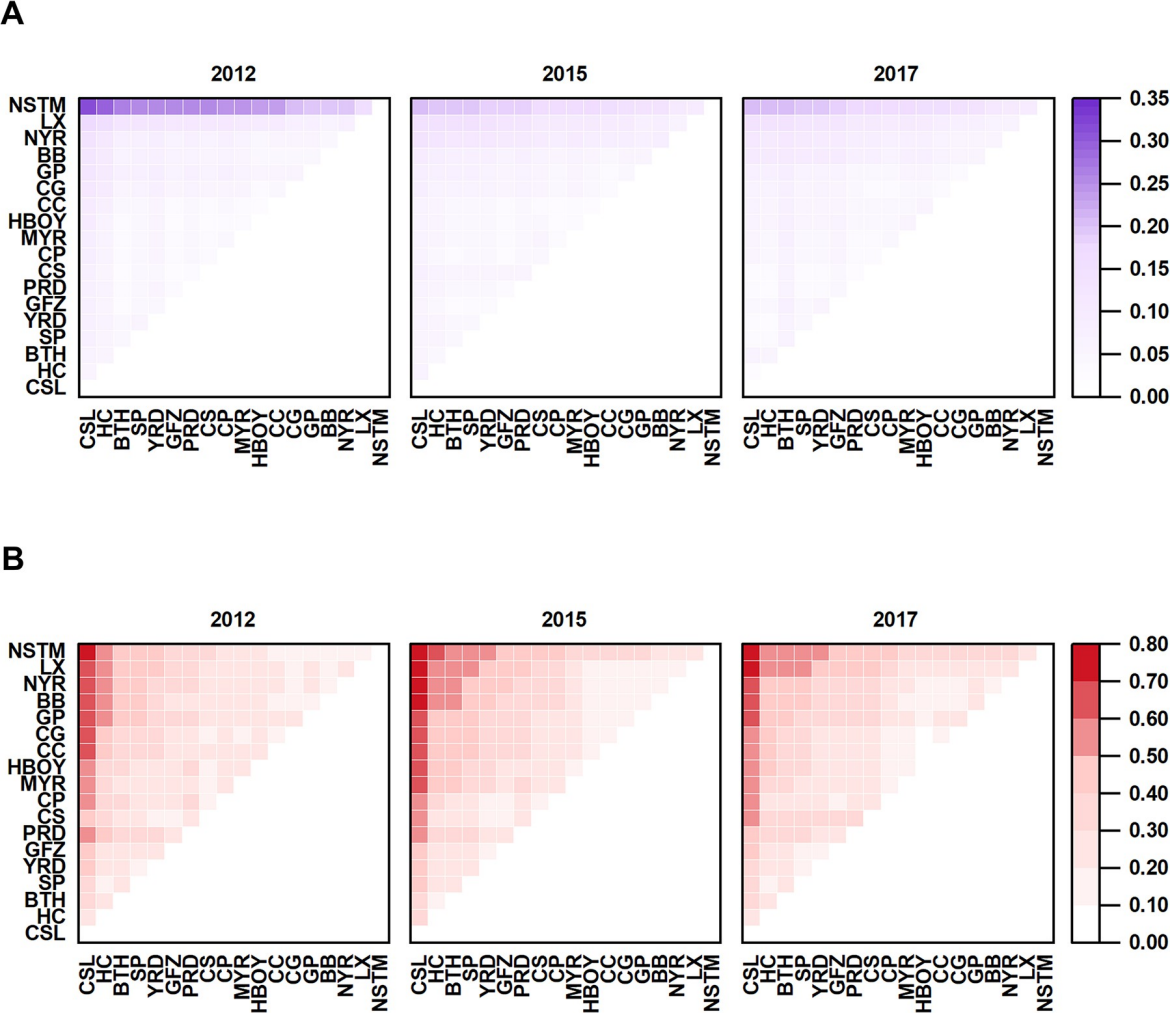
Analysis of inter-UA disparities in Chinese UAs. (A) NVC analysis. (B) GVC analysis.

Fig 2A depicts the inter-UA disparity in the NVC. Notably, NSTM is distinguished from other UAs and marked by a pronounced parallel line in the chart. This distinctly illustrates the unique role of the NSTM within the NVC, characterized by marked disparities in comparison to other UAs. Positively, there is a discernible trend of diminishing disparities over time, indicative of the NSTM’s advancing integration into the NVC. This trend is significant for fostering balanced development across Chinese UAs, as diminishing disparities in NVC among different agglomerations aid in bolstering the resilience and stability of the national economy.

Fig 2B visualizes the inter-UA disparity in the context of the GVC, revealing notable differences among the 18 UAs. A key focal point at the aggregated level of these regions is represented by a pronounced vertical line: CSL. While the CSL stands out in the GVC, the chart also displays disparities among other UAs, each with unique industrial strengths and competitive advantages. These distinctions underscore the varied characteristics and competencies of each UA within the GVC’s competitive landscape.

Drawing upon the research of Zhao et al. (2020) [48] and Yang et al. (2020) [49], the disparities among UAs can be attributed to a range of factors, including geographical location, resource endowment, economic development levels, and industrial structures. These elements not only underlie the disparities observed among Chinese UAs but also significantly influence their competitiveness in both domestic and international markets. For example, UAs with strategic geographical locations may secure more advantageous positions in the GVC, while resource-rich regions could possess heightened competitiveness in certain industrial sectors. The interplay of these factors culminates in the observed heterogeneity among Chinese UAs within the "dual value chains".

#### Sources and contribution rates of UA disparities

Table 2 discloses the pivotal dynamics of UA disparities within "dual value chains". Initially, a notable pattern emerges where the inter-UA contribution rates consistently surpass those of the combined intra-UA and super-variation density contributions. This trend suggests that disparities between UAs are the primary contributors to overall UA disparities in China. It reflects the distinct positions and competitive edges of different UAs within the new developmental paradigm, in addition to the evolving nature of their cooperative and competitive interactions. Furthermore, throughout the study period, the intra-UA and super-variation density contribution rates exhibited an upward trajectory, while the inter-UA contribution rates showed a marginal decrease. This shift points toward a more balanced development of "dual value chains" in China, albeit against a backdrop of escalating inequality within individual UAs. This dynamic indicates a multifaceted scenario in the economic evolution of Chinese UAs: intensifying cooperation and competition among different UAs on one side and, on the other, the growing disparity within them. Collectively, these factors intricately define the economic contours of Chinese UAs.

**Table 2:** Sources and contribution rates in the "dual value chains".

Value chain	Year	Intra-UA	Inter-UA	Super-variation density
NVC	2012	4.43%	77.19%	18.38%
	2015	5.58%	70.42%	23.99%
	2017	5.07%	72.81%	22.12%
GVC	2012	4.68%	76.59%	18.73%
	2015	4.47%	78.73%	16.80%
	2017	4.78%	75.07%	20.14%

### Dynamic evolution of "dual value chains" in UAs

**Fig 3:**
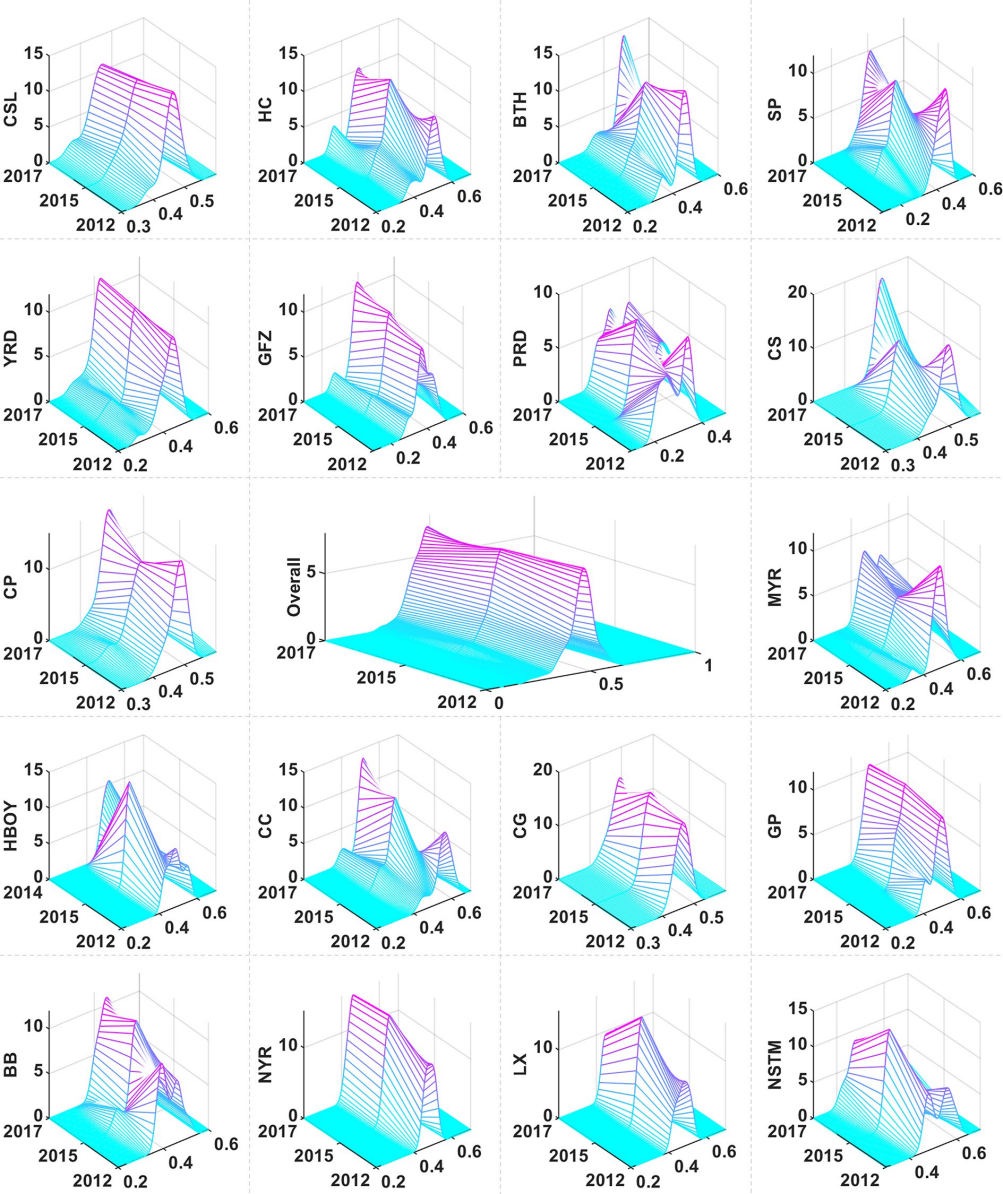
Distribution dynamics in NVC of China’s UAs.

Fig 3 depicts the evolving distribution dynamics of Chinese UAs within the NVC. A minor rightward shift in the distribution was observed, indicative of an overall enhancement in NVC engagement. This shift reflects advancements in industrial upgrading, technological innovation, and increased economic dynamism within UAs. Additionally, a decrease in both the peak height and width suggested a reduction in the concentration of UAs with high NVC integration. An increasing number of UAs are taking on significant roles in the NVC, leading to a broader distribution range. Notably, the absence of tailing and polarization in the distribution imply relatively minor disparities among UAs in the NVC, pointing to a trend toward more evenly distributed roles and statuses within the NVC and a diminution of extremes in integration levels. Collectively, these changes signify a positive evolution in the role of Chinese UAs within the NVC, reflecting both the optimization of industrial structures and a movement toward balanced development among UAs.

The observed shifts in distribution location elucidate overarching trends in NVC engagement among various UAs. The data indicate that more than 70% of UAs exhibit a rightward shift, signaling a general enhancement in NVC participation. In contrast, certain UAs, including HC, SP, YRD, CC, NYR, and NSTM, exhibited a leftward movement, denoting either a relative decrease or uneven distribution in NVC involvement. Analyzing the evolution of the primary peak reveals trends in NVC concentration or dispersion. Many UAs, such as the CS, HBOY, and GP, demonstrate an increase in peak height coupled with a narrowing width, indicating a greater concentration in NVC. In contrast, UAs such as CSL, BTH, and MYR show a reduction in peak height alongside a widening width, suggesting a dispersion in NVC involvement. For distribution ductility, most UAs exhibit minimal tailing, signifying a relative concentration in their NVC. However, HC and BTH demonstrated left tailing, while the NSTM exhibited right tailing, reflecting imbalances and dispersion tendencies in their NVC. Concerning polarization, with the exception of HC and the NSTM, the other UAs do not show significant polarization trends. The multi-polarization distributions in HC and NSTM suggest that these UAs have a more diversified and dispersed NVC distribution, with groups at varying levels of integration.

**Fig 4:**
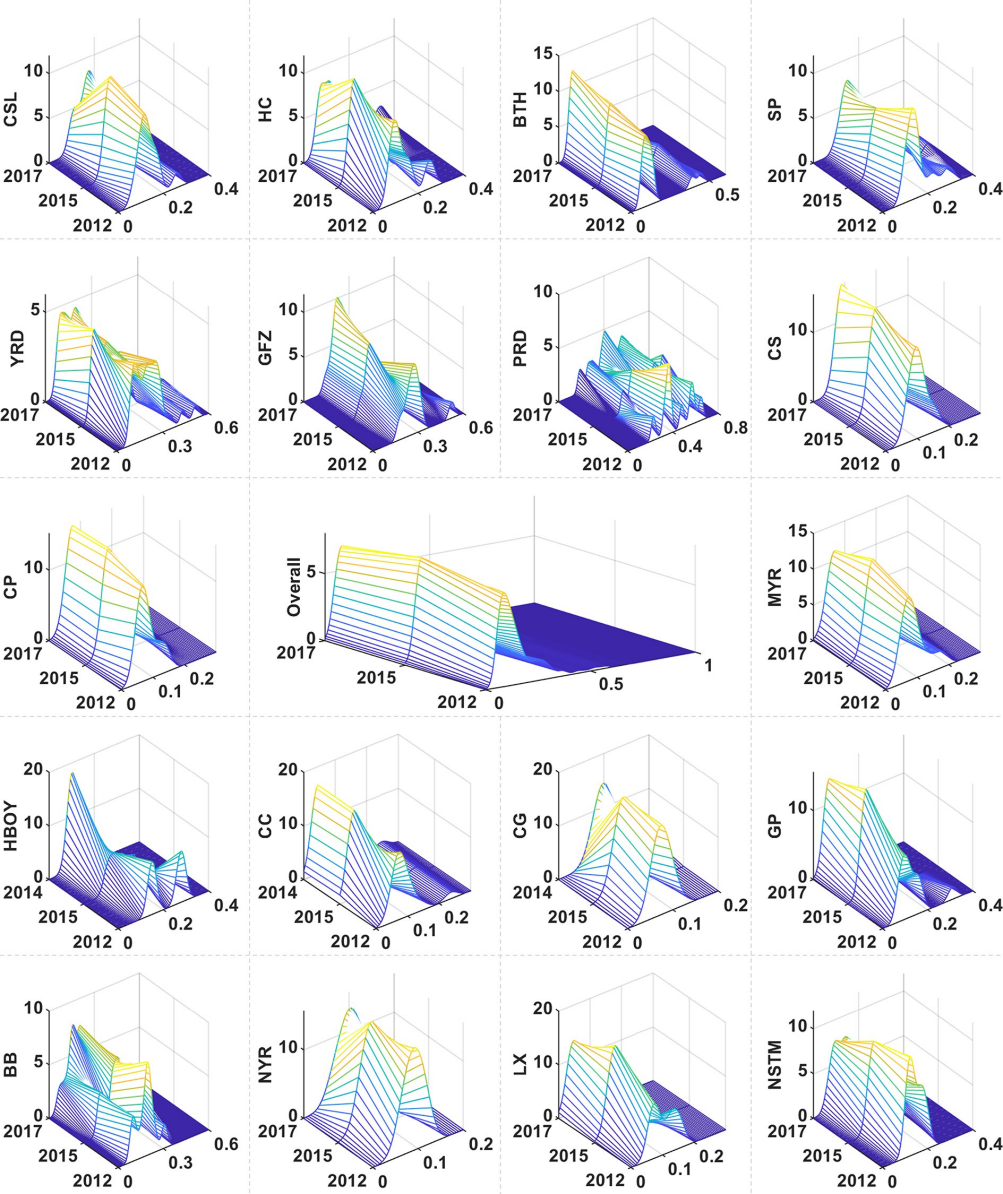
Distribution dynamics in the GVC of China’s UAs.

Fig 4 presents the dynamic evolution of Chinese UAs within the GVC. A notable leftward shift in the distribution of UAs signals a relative decline in GVC integration, which can be attributed to intensified competition in international markets, shifts in the global economic climate, and adjustments in both domestic and international policy frameworks. Simultaneously, a reduction in peak height coupled with a narrowing of distribution width indicates greater concentration of GVC integration. This pattern suggests a converging status among the majority of UAs within the GVC, with a narrowing gap between highly and less integrated UAs. Additionally, the distribution’s left-tailing characteristic and multi-polarization trends point to both an imbalance and diversification in integration levels. While the overall distribution appears relatively concentrated, significant variances persist in the level of GVC integration among certain UAs. These changes illustrate that Chinese UAs’ integration into the GVC is undergoing complex adjustments influenced by global market dynamics and domestic economic structural shifts.

Observations of distribution location shifts reveal that UAs such as CSL, SP, PRD, CG, and NYR are exhibiting a rightward trend. This symbolizes a significant enhancement in their GVC positions, stemming not only from global economic changes but also from regional industrial restructuring and upgrading. In contrast, UAs such as HC, BTH, and YRD are moving leftward, indicating a relative decline in their GVC positions due to increased global market competition and evolving domestic and international policy landscapes. The primary peak evolution in some UAs, such as the SP, PRD, and MYR, shows a decrease in peak height and an expansion in width. This implies that despite an overall rise in GVC, the number of UAs with higher GVC integration is decreasing, leading to a more diverse distribution range. Conversely, the increase in peak height and narrowing width in UAs such as GFZ, CS, and CP indicate more concentrated GVC integration, reflecting their leading roles in the global economy. Regarding distribution ductility, most UAs showed no significant tailing, suggesting a more concentrated GVC distribution. However, the right tail observed in UAs such as BTH, GFZ, and PRD indicates an imbalance and dispersion in the GVC distribution. While most UAs do not exhibit notable polarization trends, the multiple polarizations observed in clusters such as YRD, GFZ, and CC suggest a more dispersed GVC distribution, indicating variances at different levels of GVC integration.

### Contributions of drivers

Since NVC and GVC play a crucial role in the participation of Chinese UAs in the new development paradigm, we consider NVC/GVC as driver, while also taking into account three other drivers (OE, PGDP, and POP) that determine NII/GII. Then, we apply the LMDI method to decompose and quantify the contribution of each driver to the variations in NII and GII from 2012 to 2017.

**Fig 5:**
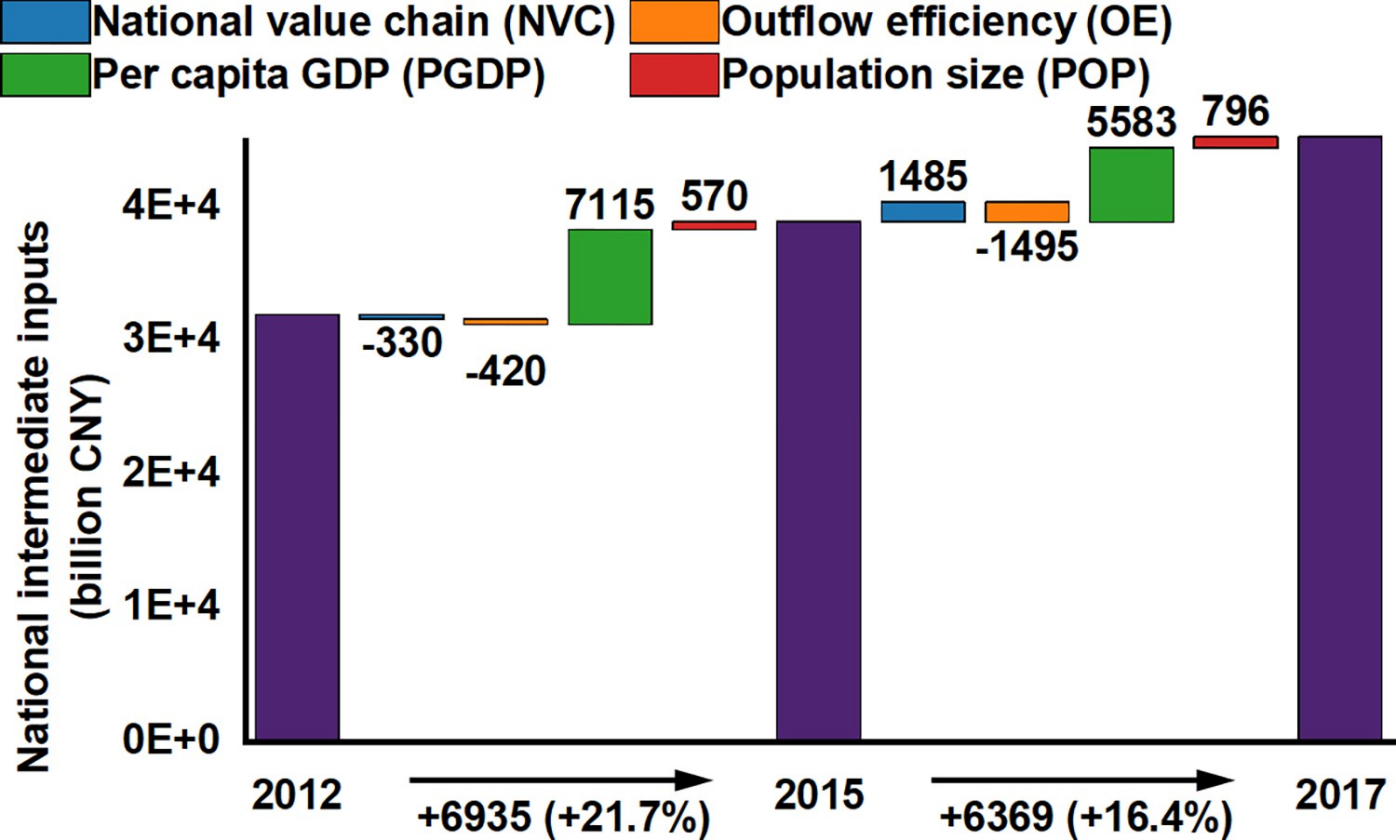
Four drivers of variation in the NII of China’s UAs from 2012 to 2017. The values beneath the bar chart represent the variation in the NII of China’s UAs between the two periods. The dark purple bars display the total annual NII of China’s UAs.

In the LMDI analysis examining changes in NII of UAs between 2012–2015 and 2015–2017, distinct economic patterns emerged, as depicted in Fig 5. Between 2012 and 2015, UAs grappled with two challenges: a contraction in the NVC and a reduction in OE. The negative growth in NVC (-330 billion CNY) mirrors substantial industrial restructuring. Traditional industries experienced a decline, while emerging sectors had not yet fully matured to compensate for this downturn. This transformation signifies the economy’s shift from rapid expansion to a "new normal", emphasizing efficiency enhancement and structural optimization. The "new normal" encapsulates China’s evolving economic model, aspiring for higher-quality growth that is sustainable, both economically and environmentally, and yields improved social outcomes. Concurrently, the decline in OE (-420 billion CNY) denotes an increase in regional factor retention, largely owing to increased local investments and advancements in the industrial chain. This bolstered factor retention has propelled the growth of nascent industries and enhanced production efficiency, pivoting the economy toward high-efficiency operations and quality-focused capital deployment. Moreover, the notable increase in PGDP (+7115 billion CNY) and POP growth (+570 billion CNY) during this period underscore an increase in economic productivity and a burgeoning labor market.

In the subsequent period of 2015–2017, the trends diverged from the earlier phase. The marked positive growth in NVC (+1485 billion CNY) exemplifies enhanced production efficiency and the pivotal influence of technological innovation in augmenting national value-added. This upswing is principally attributed to progress in automation, digitization, and more efficient production methodologies. Furthermore, escalating internal market demand, encompassing consumer expenditure and corporate investments, significantly spurred economic activity. In contrast, the continued negative growth in OE (-1495 billion CNY) signals the optimization of regional economic structures and a transition toward an innovation-led economic paradigm. This indicates more effective local resource utilization and a diminishing reliance on external resources. However, the decline in PGDP growth (+5583 billion CNY) presents a challenge, suggesting that the current economic expansion is leaning more on labor growth than on productivity improvements. This could imply either a deceleration in technological advancement or inadequate investment in human capital. Nevertheless, consistent POP growth (+796 billion CNY) reflects the enduring allure of UAs for talent, a vital element in sustaining labor market vibrancy and supporting overall economic growth.

**Fig 6:**
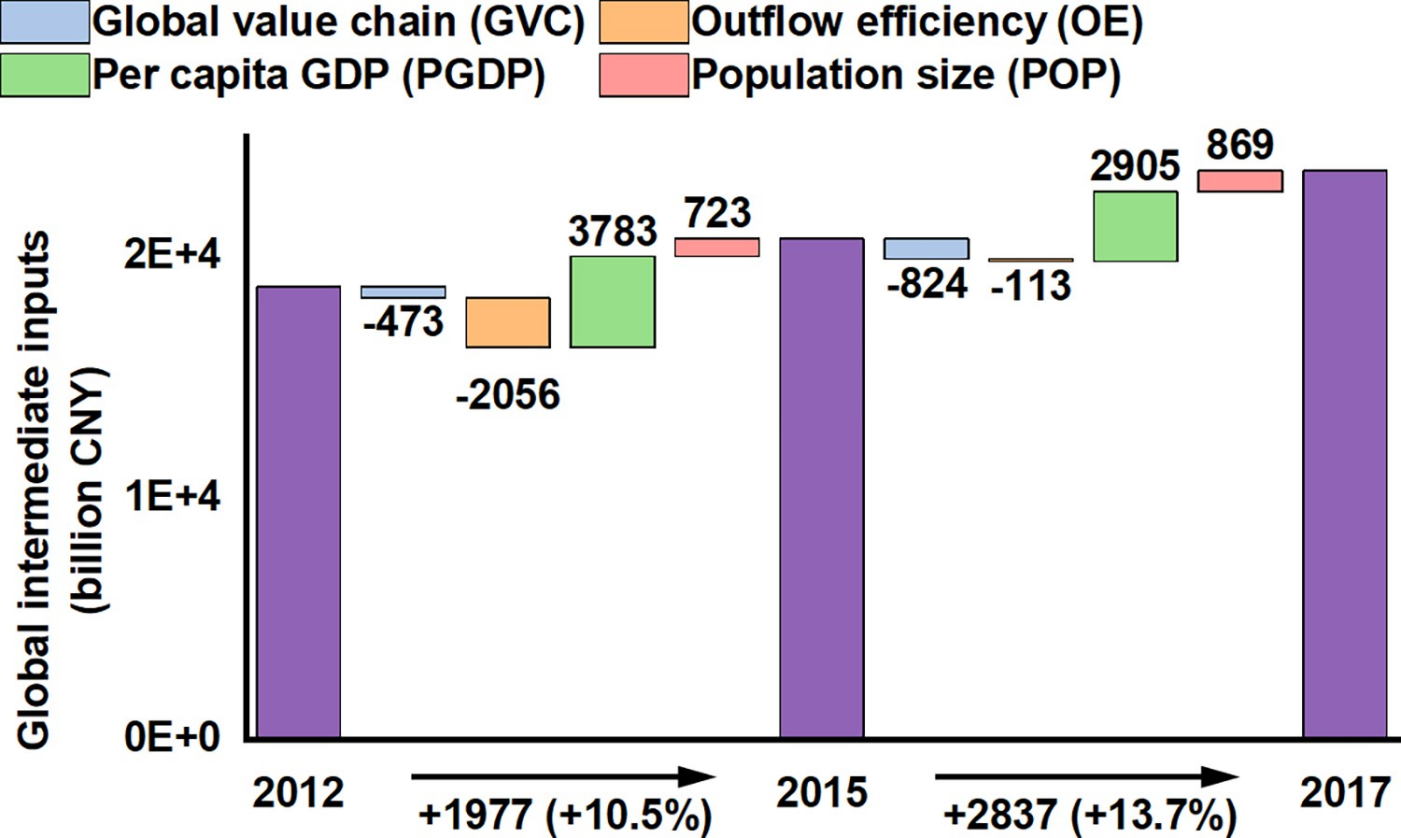
Four drivers of variation in the GII of China’s UAs from 2012 to 2017. The values below the bar chart detail the variation in the GII of China’s UAs across the two periods, with the light purple bars representing the total annual GII of China’s UAs.

Fig 6 presents the LMDI decomposition of the total GII for UAs across 2012–2015 and 2015–2017. From 2012 to 2015, Chinese UAs witnessed a contraction (-473 billion CNY) in the GVC. This downturn in the GVC signifies a decrease in fund flows and economic vitality within the domestic cycle, influenced by domestic macroeconomic regulatory policies such as deleveraging and structural adjustments. These policies temporarily subdued capital flow vitality, reflecting the challenges of economic transition. Simultaneously, a marked decline in OE (-2056 billion CNY) mirrored the challenges faced by the export-oriented economy, including reduced global trade demand and a relative dip in the competitiveness of Chinese products internationally. Fluctuations in the RMB exchange rate, heightened international trade barriers, and rising domestic production costs also contributed to this decline. Contrasting these challenges, the substantial increase in PGDP (+3783 billion CNY) indicated qualitative growth in UAs’ economies, driven by improved labor productivity and the development of high-tech industries and services. This underscores the resilience of UAs’ economies amid transformation and upgrading. Steady POP growth (+723 billion CNY) mirrors the ongoing urbanization process and the beneficial impact of economic development on population attraction and is vital for supporting sustained economic growth in UAs.

In 2015–2017, the negative trend in GVC for Chinese UAs intensified (-824 billion CNY), raising concerns about the depth of China’s macroeconomic adjustments and global economic uncertainties. This downturn was primarily due to a slowdown in domestic investment and consumption, compounded by persistent global trade tensions. However, the reduced negative growth in OE (-113 billion CNY) suggests that export markets are adapting to the new international trade environment, striving to regain momentum by optimizing export structures and enhancing product competitiveness. Despite a slowdown in PGDP growth to +29051 billion CNY, the continued positive trajectory indicates efficiency improvements and ongoing quality enhancement in economic activities. Concurrently, accelerated POP growth (+869 billion CNY) reflects, to an extent, the hastening of urbanization and the sustained draw of UAs for talent and the labor market.

## Conclusions, implications and limitations

### Conclusions

UAs, which centralize China’s considerable population and economic activities and exert substantial influence, hold a unique position in shaping new development paradigm. This study aimed to quantify the integration of UAs into this new paradigm from 2012 to 2017, seeking to offer insights into their functional role and strategies to mitigate regional imbalances. The principal research findings are summarized as follows:

1. Throughout the analysis period, Chinese UAs gradually transitioned from integrating into the GVC to more prominently integrating into the NVC within the new development framework. Specifically, the spatial distribution of NVC exhibited a pattern of "low in the east, high in the west, and evenly distributed in the north and south". In contrast, the GVC exhibited a distribution of "high in the east, low in the west, low in the north, and high in the south".
2. In terms of relative disparities, the contribution rates of inter-UA disparities consistently exceeded the combined rates of intra-UA and super-variation density contributions, albeit showing a gradual declining trend. The overall disparity within the NVC and the inter-UA disparity both narrowed. Moreover, the overall disparity in the GVC, as well as the inter-UA disparity, initially increased but later decreased. The intra-UA disparity within the "dual value chains" demonstrated significant heterogeneity.
3. From a distribution dynamics perspective, the NVC in most UAs showed a rightward shift, with an increase in peak height, no apparent trailing phenomena, and no polarization trends; additionally, the width changes were average. For the GVC in most UAs, the distribution location displayed a leftward shift without obvious trailing phenomena. The peak evolution presented complex patterns, with both multi-polarization and nonpolarized trends being equally prevalent.
4. Regarding the driving factors, an increase in the PGDP was the primary contributor to the growth of NII, and POP size expansion also had a significant positive impact on NII. The NVC changed from negative to positive, emerging as the second most important driving factor during 2015–2017. OE exhibited a negative driving effect with an increasing trend. An increase in PGDP and POP size exerted substantial positive driving effects on GII. While the GVC and OE had negative driving impacts, their combined negative influence showed a decreasing trend.

### Policy implications

Considering the pivotal role of UAs in China’s new development paradigm, alongside the existing conditions, disparities, and evolutionary traits of the "dual value chains" within these UAs, a comprehensive set of policy recommendations is presented. These suggestions aim to optimize the NVC, enhance the GVC, and balance the "dual value chains":

#### For optimizing the NVC

Firstly, by emphasizing the importance of leveraging policy support and incentive mechanisms to foster economic, technological, and resource sharing among UAs. This approach not only facilitates complementary and cooperative interactions among regional industries but also aims to mitigate developmental imbalances, thereby encouraging synergistic regional growth. Furthermore, prioritizing investments in and the upgrading of essential infrastructure, including transportation and information and communication technologies, is proposed to bolster the efficient flow of logistics, capital, and information both within and across UAs. This, in turn, is expected to strengthen infrastructure connectivity. Lastly, the study advocates for the promotion of high-tech industries alongside supporting the technological transformation and upgrading of traditional sectors. This dual strategy is anticipated to accelerate the shift towards an innovation-driven development model, contributing to the optimization and advancement of the industrial structure.

#### For enhancing the GVC

Firstly, UAs should prioritize increasing investments in research and development (R&D) to spur innovation within key sectors. This entails the development of new technologies, products, and processes with the aim of securing a competitive edge in the global value chain. Furthermore, it is essential to upgrade industrial infrastructure and integrate advanced manufacturing technologies and services, such as automation, digitalization, and smart manufacturing, to enhance production efficiency and product quality. Lastly, UAs must also focus on improving the education and training system to bolster the skills and professional competencies of the workforce, thereby raising the overall quality of the population. This comprehensive approach supports industrial innovation and strengthens global competitiveness.

#### For balancing the "dual value chains"

Firstly, establishing a more open and dynamic economic framework is crucial for optimizing the structure of foreign trade and achieving balanced development between domestic and international markets. This strategy significantly enhances the economic system’s adaptability and responsiveness to global economic fluctuations. Additionally, it is vital to fortify the stability of both industrial and supply chains. Achieving this stability involves diversifying sources of imports and expanding domestic production capabilities, which, in turn, diminishes reliance on external shocks and bolsters the resilience and autonomy of the supply chain. Lastly, prioritizing green and low-carbon development is imperative. Recognizing the shift towards sustainability as a pivotal strategy to boost the competitiveness of the "dual value chains" is fundamental. Promoting the use of environmental technologies and products, alongside improving resource efficiency, is instrumental in driving sustainable development.

These recommendations are designed to bolster the internal development and external collaboration capabilities of UAs, thereby enhancing their roles in both the domestic and international value chains. This strategy aims to drive the high-quality development of China’s economy and improve its integration into the global economic system.

### Limitations and future research

This study provides recommendations for achieving balanced development in Chinese UAs. Further incorporation of emerging economic trends, such as digitalization and sustainability, into the analysis would offer a more forward-looking perspective on the development trajectories of UAs. Additionally, due to data limitations, our research only analyzed UA disparities from 2012 to 2017. To provide more timely policy recommendations, integrating a broader range of data sources and extending the analysis period beyond 2017 is necessary.
